# Evaluation of Aroma Compounds in the Process of Wine Ageing with Oak Chips

**DOI:** 10.3390/foods8120662

**Published:** 2019-12-10

**Authors:** Georgiana-Diana Dumitriu (Gabur), Carmen Teodosiu, Iulian Gabur, Valeriu V. Cotea, Rafael A. Peinado, Nieves López de Lerma

**Affiliations:** 1 Department of Environmental Engineering and Management, Gheorghe Asachi Technical University of Iasi, 700050 Iasi, Romania; diana.gabur@tuiasi.ro; 2 Department of Plant Science, Ion Ionescu de la Brad University of Agricultural Sciences and Veterinary Medicine of Iasi, 700490 Iasi, Romania; 3 Department of Viticulture and Oenology, Ion Ionescu de la Brad University of Agricultural Sciences and Veterinary Medicine of Iasi, 700490 Iasi, Romania; vcotea@uaiasi.ro; 4 Agrifood Campus of International Excellence ceiA3, Department of Agricultural Chemistry, University of Córdoba, 14014 Córdoba, Spain; qe1peamr@uco.es

**Keywords:** American and French oak chips, red wine, SBSE–GC–MS, aroma compounds, heatmap, sensorial analysis

## Abstract

Many modern alcoholic beverages are subjected to ageing processes during which compounds extracted from wood contribute decisively to the overall beverage character. Wines represent a perfect example of beverage in which ageing is a crucial technological manufacturing step. During winemaking, producers accelerate chemical changes in wine composition by traditional and alternative methods, such as the use of oak wood barrels and/or oak wood chips. Our research aimed to investigate the overall volatile composition and sensory quality of red wines aged for two timeframes, namely, 1.5 and 3 months, and with two technological variants, i.e., American and French oak wood chips. Red grapes from the Fetească neagră (*Vitis vinifera*) variety were harvested from a vineyard in the North-East region of Romania. Stir bar sorptive extraction and gas chromatography coupled with mass spectrometry (SBSE–GC–MS) was used to extract minor aromas present in wine samples. The results showed clear differences between wines treated with American and French oak chips. Furthermore, ageing for 3 months increased the concentration of *cis*-whiskey lactone and guaiacol in American oak-treated wine samples. For wines aged with French oak chips, we observed higher concentrations of furfural, 5-methylfurfural, 4-vinylguaiacol, and *trans*-whiskey lactone. The increased presence of chemical compounds in wine aged with French oak chips generated prominent smoky, licorice, and toasty aromas, whereas in wines aged with American oak chips, notes of vanilla, toasty, and cacao aromas were noticed. Moreover, red wines aged with American and French oak chips were discriminated by chemometric analysis, which confirmed the evolution of aroma compounds.

## 1. Introduction

The ageing process, in traditional oak wood barrels or by using modern/alternative oak wood chips, plays an indispensable role in the manufacturing of high-quality and typical red wines. Oak wood ageing methods causes particular changes in red wines chemical composition, leading to spontaneous clarification, lower astringency, decrease of excess vegetative notes, and achievement of new complex aromas known as “oak aromas”.

Even if there are over 150 different species of oaks ranked in the genus *Quercus*, in winemaking, only three are mostly used for wine ageing [1]. More precisely, these are the American white oak (*Quercus alba*) and two European oaks, the sessile oak (*Quercus petraea*) and the pedunculate oak (*Quercus robur*), commonly known as the French oaks. Since wine ageing in barrels is expensive and requires long time periods, the utilization of oak chips is a valid alternative for reducing production costs and accelerating wood-flavored winemaking. Also, in winemaking, oak wood chips allow ageing in stainless-steel containers, as it conveys similar tastes and aromas to wines aged in barrels but without the large number of drawbacks: short time usage, difficult temperature control, barrel cleaning, presence of undesirable microorganism such as *Brettanomyces*/*Dekkera*, wine loss due to evaporation, negative environmental impacts caused by deforestation, high economic impacts, etc. The European Union (Council Regulation EC No. 2165/2005) and the International Vine and Wine Organization (OIV, Resolution OENO 3/2005) authorized the use of alternative oak products in winemaking, which resulted in an increased demand of oak chips and staves [2].

Oak species, geographical origin, morphological characteristics of oak wood (granularity, porosity, and permeability), processing and chemical composition, dosages of chips, type of toast, and contact time influence the sensible physio-chemical and biochemical mechanisms that occur during the ageing process. Oak chips are known to affect wine chemical composition and overall sensory properties and contribute to their stability [3]. Phenolic and volatile compounds are exposed to transfer from oak wood to wine. These aspects emphasize the importance of selecting appropriate oak woods, dosages, and contact time between wine and chips for obtaining the desired phenolic and volatile composition.

Among the volatile compounds found in oak wood, lactones, especially *cis*- and *trans*-methyl-octa lactones, are known for their remarkable sensorial impacts in red wines. They are key compounds in ageing due to their low perception threshold and oak or coconut characters. Vanillin and its many derivatives are described in the literature as key contributors of the vanillin flavor, while small free phenolic compounds such as eugenol and guaiacol are characterized by spice, clove, and smoke notes [4]. In contrast, furfural and 5-methylfurfural have been previously indicated as sources of almond and toasted almond aromas. However, furan derivatives are not considered major contributors to red wine aromas due to their high perception threshold levels, but they may enhance the perception of other oak compounds, such as lactone. Generally, oak volatile concentration increases in wines proportionally to the ageing time. Exception to these rules are furfural and 5-methylfurfural which accumulate in the first wood–wine contact time period but are afterwards reduced to their corresponding alcohols [5].

Until now, the volatile compounds and sensory characteristics of Romanian red wines aged with American and French oak chips have not been studied. In the literature, there are some studies on aroma compounds in wines aged using alternatives techniques [6,7]. Cabrita et al. [8] investigated the furanic derivatives, phenolic acids, and phenolic aldehydes present in oak wood chips, comparing American oak and French oak. Their results indicated that the wine aged with French oak wood chips is richer in aroma compounds than the wine aged with American oak chips. Moreover, French oak chips contribute to increased levels of furanic aldehydes in wines. As reported by various authors, the concentration of *cis*-lactone, which is known as an important aroma compound, is higher in American oak in comparison to French oak [9]. Consequently, oak chips should be chosen with great attention by looking at the entire characteristics of the wood. In winemaking, dosing of oak fragments is not an easy task, as various factors, especially, quantity and contact surface, influence the characteristics of the final product.

The diversity of alternative oak fragments allows oenologists to achieve varying results depending on the expected outcome and consumer preferences. Prolonged exposure to oak wood may cause unbalanced flavor profiles with strong oak aromas.

Volatile compounds extraction procedures depend on the development of high-sensitivity, accurate, and automated methodologies that can reduce the error rate and sample handling time. Stir bar sorptive extraction (SBSE) is a new, cost-efficient, and reliable method to deal with liquid samples, in which a stir bar coated with polydimethylsiloxane (PDMS) is used to extract analytes directly from treated matrices [10]. PDMS is nowadays the most frequently used polymer because of its robustness and stability. Recent literature reports described SBSE-based techniques for the detection of volatile compounds in wine samples [11,12,13].

Romania is an important European wine producer, with a strong history of winemaking and an increasing interest for autochthonous wines. One of the favorite local red wine varieties of Romanian consumers is obtained from Fetească neagră (*Vitis vinifera*) grape, according to the International Vine and Wine Organization (OIV) reports. This variety is an old Romanian–Moldavian dark-skinned grape variety developed in the Uricani, Iasi, winemaking region. Fetească neagră aged wine is considered one of the best red wines due to its smoky, plum, black currant, and chocolate characters. According to the OIV, in 2017, 3000 ha and 1.6% of the total vineyard area of Fetească neagră have been cultivated.

The purpose of the present study was to examine the effects of different types of oak chips, dosages, and ageing times on wine composition by using gas chromatography coupled to mass spectrometry (GC–MS) in combination with analyte enrichment techniques, such as SBSE. Besides, we focused on identifying the chemical compounds and technological parameters that may discriminate wine samples according to contact time, wood type, or dosages. This study provides important scientific content with practical benefits to winemakers. Upgrading the winemaking stages with alternative ageing methods and enhanced control on the wine–wood extraction process will contribute to high-quality wine production.

## 2. Materials and Methods

### 2.1. Winemaking Process

*V. vinifera* cv. Fetească neagră red grapes were obtained from the North-East Romania (Șuletea, Vaslui) winemaking region and were harvested in their optimal ripening stage in good sanitary conditions, in 2013. The wines were processed in a pilot-scale installation of the Oenology Department of “Ion Ionescu de la Brad” University of Agricultural Sciences and Veterinary Medicine, Iaşi, following the traditional red winemaking process. The marc obtained after crushing the grapes was subjected to maceration–fermentation at 10–12 °C, for a period of 7 days. The marc was pressed using a pneumatic press, Vaslin–Bucher XPRO 5, France, and the wine obtained was transferred to fermentation tanks for completing the alcoholic and malolactic fermentation. The commercial active dry yeasts Fermactive^®^ Rouge Primeur and Fermactive^®^ Activateur Complex (Sodinal) were used. Wines were supplied with 60 mg/L of sulphur dioxide and divided in 15 stainless-steel tanks of 250 L. Each experimental variant was reproduced in triplicate, generating in total 15 tanks of 250 L (3 tanks for Control Wine aged without oak chips, 3 tanks for wines aged with American oak chips (3 g/L), 3 tanks for wines aged with French oak chips (3 g/L), 3 tanks for wines aged with American oak chips (5 g/L), and 3 other tanks aged with French oak chips (5 g/L). At the end of the fermentation processes, the wine samples were supplied with oak chips at dosages of 3 g/L or 5 g/L. The oak chips were obtained from medium-toasted American oak (*Q. alba*) and French oak (*Q. petraea*), in accordance with the Council Regulation EC No. 2165/2005. Am3 and Am5 represent the samples aged with American oak chips, Fr3 and Fr5 represent the samples aged with French oak chips. The wines were aged for two time periods, i.e., 1.5 and 3 months. The chips dimensions in centimeters were 0.5 × 1.5 × 0.2 (width × length × thickness). The wines were filtered, bottled, and stored for 6 months in a conditioned room at 10 °C. Furthermore, wine samples were selected for chemical and sensory analysis.

### 2.2. Standard Chemical Analysis of Wine

O.I.V. International Oenological Codex [14] was used for the analysis of pH, total and volatile acidity, alcoholic strength (% *v*/*v*), reducing sugar, total and free SO_2_ in Fetească neagră red wines. Malolactic fermentation was controlled by monitoring the L-malic acid amount of the wines. Malic acid content reached values ≤0.2 g/L, controlled every two days by an enzymatic test kit. In order to characterize the total phenol index (TPI), we used a spectrophotometric measurement at 280 nm (OIV). The absorbance at 420, 520, 620 nm and CieLab parameters (L *, a *, b *) were determined in a Perkin-Elmer Lambda 25 spectrophotometer (MA, USA), after filtering the samples through a HA-0.45 μm paper (Millipore, Milford, MA, USA).

### 2.3. Minor Aroma Compounds

#### 2.3.1. Extraction of Minor Aroma Compounds

The extraction of minor aroma compounds was carried out in accordance with Lopez de Lerma et al. [15]. A stir bar (0.5 mm thickness; 10 mm length; Gerstel GmbH, Mülheim an der Ruhr, Germany) coated with PDMS was used. All chemicals were of analytical grade and were supplied by Merck and Sigma Aldrich. The samples were diluted in a proportion of 1:10 with a hydro ethanolic solution that contained 12% ethanol (*v*/*v*) and which was previously adjusted to pH 3.5 with 2.6 g/L tartaric acid and 2.2 g/L potassium bitartrate. The stir bars were placed in a 10 mL glass headspace vial with 10 mL of the diluted sample and 0.1 mL of a solution of ethyl nonanoate (0.4464 mg/L) as internal standard. Teflon-coated crimp caps were used to seal the vial. The stir bar was stirred at 1500 rotations per minute at 25 °C for 100 min. The wine sample were removed, and the stir bar was gently dried with a lint-free tissue and then transferred into a glass thermal desorption tube for GC–MS analysis.

#### 2.3.2. Determination of Minor Aroma Compounds

A GC–MS equipped with a Gerstel TDS 2 thermal desorption system was used. The glass thermal desorption tube was introduced gently in the GC–MS. Firstly, the stir bar was heated to release and transfer the extracts into a cooled injection system/programmed temperature vaporizer (CIS 4 PTV) containing a Tenax adsorption tube. Thermal desorption was carried out following the program: 35 °C, ramped at 120 °C min^−1^ to 280 °C, and held for 10 min; the helium flow rate was 3 mL/min. The CIS injector was held at 25 °C for the total desorption time and then ramped at 12 °C s^−1^ in splitless mode to 280 °C and held for 7 min.

The GC was equipped with an Agilent-19091S capillary column (30 m × 0.25 mm, 0.25 µm thickness, Santa Clara, CA, USA) Helium was used as carrier gas with a column flow rate of 1 mL min^−1^. The GC was programmed as follows: 50 °C for 2 min, ramped at 4 °C min^−1^ to 190 °C, held for 10 min. The mass detector was used at 1850 V in the scan mode, and the studied mass range spanned values from 39 to 300 *m/z* (represents mass divided by change number and the horizontal axis in a mass spectrum). The operation conditions for GC and MSD (mass selective detector) were detailed by Lopez de Lerma et al. [15].

Retention times, spectral libraries supplied by Wiley (version 7 N), and pure chemical compounds obtained from Merck, Sigma–Aldrich, Riedel de Haën, and Fluka were used for identification, confirmation, and preparation of standard solutions of the volatile compounds. The compounds were quantified from their calibration curves, which were obtained by using standard solutions at known concentrations previously subjected to the same treatment as the samples, in conjunction with the target and qualifier ions selected for each compound by the Hewlett–Packard Chemstation (Palo Alto, CA, USA).

### 2.4. Sensory Analyses

The effect of the ageing process on the sensory characteristics of the red wines was evaluated by subjecting various samples to a detailed sensory analysis. Wines, previously kept in cold storage, were transferred to room temperature (20 °C) before the sensory analysis. The evaluation panel was composed of 15 tasters (6 women and 9 men) between 25 and 60 years of age. All tasters had a winetasting degree and experience. Also, some members of the sensory panel are actively involved in the International Organization of Vine and Wine. The objective and methodology of each test were explained before the start of the sensory analysis.

Specific aroma descriptors were used: ripe fruit, herbaceous, woody, toasty, smoky, vanilla, caramel, cacao, spice, and licorice. The tasters graded each specific aroma descriptor with a score from 1 to 5 (1—detectable, 2—weakly perceptible, 3—moderately perceptible, 4—strongly perceptible, and 5—very perceptible) in increasing order of perceived intensity.

### 2.5. Statistical Analyses

Multifactorial variance analysis was performed by using as factors the ageing time, the type of oak chips, and the dosage of chips. It determined which factors had a statistically significant effect on the amount of volatile compounds. Statistical tests were done with the software package Statgraphics Centurion XVI StatPoint Technologies, Inc. (Warrenton, VA, USA).

For the principal component analysis (PCA), the concentrations of 7 key compounds were used to build the data matrix for American and French oak chips. The values of the two time periods values pooled and used as factors for each key compound. Furthermore, a heatmap visualization of the dataset (4 American oak chips, 4 French oak chips, and 37 volatile compounds) was also obtained using XLSTAT (Addinsoft, Paris, France, version 2017, 30 days trial version).

All analyses were done in triplicate, and the results are presented as the mean values and standard deviations.

## 3. Results

### 3.1. Chemical Composition of Wines Aged with American and French Oak Chips

The main oenological parameters of wines treated with American and French oak chips after 1.5 and 3 months presented the usual values, consistent with those expected for Fetească neagră red wines from the North-East Romania region [2]. The analytical parameters of the control wine (CW) sample aged without oak chips were: ethanol (% *v*/*v*): 15.0 ± 0.2; reducing sugar (g/L): 2.36 ± 0.02; volatile acidity (g acetic acid/L): 0.48 ± 0.02; titratable acidity (g tartaric acid/L): 5.89 ± 0.04; pH: 3.68 ± 0.01; free SO_2_ (mg/L): 39.42 ± 2.5; total SO_2_ (mg/L): 113.42 ± 7.3; TPI (AU 280): 3.13 ± 0.04; *L* *: 24.49 ± 3.2; *a* *: 46.89 ± 5.5; *b* *: 28.01 ± 3.1; A420: 2.048 ± 0.02; A520: 0.127 ± 0.1 and A620: 0.706 ± 0.03.

Red wines aged with oak chips showed a high alcohol content, which was not dependent on the type and dosage of oak chips. Th results showed that the total SO_2_ amounts were below the legal limits indicated by OIV. During ageing, wines treated with oak chips presented lower amounts of tartaric acid and, on the other hand, a slight increase in acetic acid content. Thus, the total and volatile acidity were significantly dependent on ageing time and type and dosages of oak chips. The values of TPI for wines treated with American oak wood and French oak wood chips decreased after a long ageing period (3 months), and at the same time, the wine treated with French oak chips presented a higher TPI value in comparison with the wine treated with American oak chips (as depicted in Table 1). In agreement with Kyraleou et al. [16], TPI progressively decreased during ageing, possibly due to the transformation of phenolic compounds into more condensed chemical constituents with different properties and reactivity.

It was observed that the type of oak chips affected the color in the case of wines aged with American and French oak chips (Table 1). The wines aged with French oak chips presented a higher value of yellow (A420), red (A520), and blue (A620) than the wines aged with American oak chips. The yellow and blue components increased significantly with the ageing time, whereas the red component decreased with the ageing time. The condensation between anthocyanins and tannins, as well as co-pigmentation, led to the formation of new stable pigment compounds that provided a greater intensity of color, which was more resistant to degradation and characterized by increased blue tones. He et al. [17] described the formation of new stable pigments with greater intensity as a result of the condensation between anthocyanins and tannins and co-pigmentation. Similarly, Martínez-Gil et al. [18] suggested that micro-oxygenation leads to the formation of new stable pigment compounds and more intense blue tones. These parameters were significantly dependent on ageing time and type and dosages of the oak chips.

### 3.2. Volatile Profile of Aged Wines with American and French Oak Chips

SBSE–GC–MS analysis of red wines after 1.5 and 3 months of ageing with American and French oak chips identified eight chemical groups: alcohols, carbonyls, carboxylic acids, esters, lactones, terpenes, volatile phenols, and oak compounds (Table 2). Multivariate analysis was used to determine the influence of factors such as ageing time, type of oak species, and dosage of oak chips on volatile aroma.

**Figure 1:**
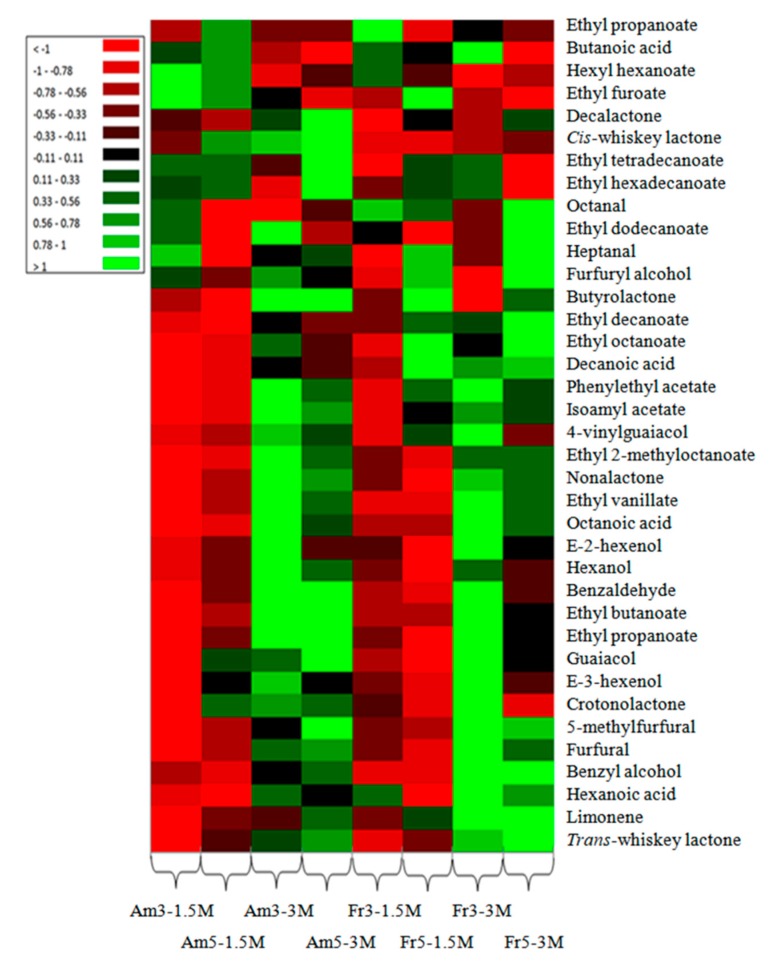
Heatmap representation of stir bar sorptive extraction (SBSE)–GC–MS-determined concentrations of minor volatile compounds in wines aged 1.5 and 3 months with American and French oak chips.

The minor volatile profile of the American and French oak chips data set was used to generate a heat map representation (Figure 1) for a rapid visual assessment of the similarities and differences between wine samples. Certainly, visible changes in volatile compounds correlated with ageing process were observed, suggesting that the wines treated with oak chips had different profiles. During ageing, specific compounds are transferred into wines as a result of the long contact with the oak chips. Two dosages of American and French oak chips were used (3 and 5 g/L), and the allowed contact time was of 1.5 and 3 months. As a consequence, each sample of wine evolved in a different manner, with visible increases of the volatile compounds in wines aged 3 months with either American or French oak chips.

As it can be observed, not always a higher chips dosage corresponded to a higher volatile compounds’ concentration. For instance, alcohols (hexanol, E-2-hexanol), carboxylic acids (octanoic acid, decanoic acid, butanoic acid), volatile phenols (4-vinylguaiacol), and oak compounds (*cis* and trans-whiskey lactone) presented higher concentrations in samples treated for 3 months with 3 g/L of oak chips than in samples treated with 5 g/L of oak chips for the same time. Other compounds such as furfural and 5-methylfurfural showed greater concentrations in samples treated with 5 g/L of American oak chips and 3 g/L of French oak chips after 3 months of ageing, whereas isoamyl acetate, ethyl octanoate, and ethyl decanoate presented higher concentrations in sampled treated with 3 g/L of American oak chips and 5 g/L of French oak chips after 3 months of ageing. These differences can be ascribed to the different composition of French and American oak [19] or to the fact that a smaller dosage of oak chips can transfer specific chemical compounds into wines more quickly. This can be caused by increased wine–wood contact that facilitates the process of aroma compound diffusion [20].

#### 3.2.1. Alcohols

Generally, alcohols are the product of the yeast fermentation of sugars and the yeast metabolism of amino acids. A recent report suggested that higher alcohols have an important impact on the fruity aroma of wine [21]. By using the SBSE–GC–MS analysis, we detected 10 alcohols, consisting of 5 major alcohols (propanol, 2-methyl-1-butanol, 2-phenylethanol, isobutanol, methanol) and 5 minor alcohols (hexanol, E-3-hexenol, E-2-hexenol, furfuryl alcohol, and benzyl alcohol) (Table 2). In the major group, 2-methyl-1-butanol was the prominent alcohol, since the concentrations found were 531 mg/L (3 g/L American oak chips), 534 mg/L (5 g/L American oak chips), 537 mg/L (3 g/L French oak chips), and 538 mg/L (5 g/L French oak chips) at 1.5 months. After 3 months of ageing, the concentration of this alcohol increased to 553 mg/L (3 g/L American oak chips), 571 mg/L (5 g/L American oak chips), to 539 mg/L (3 g/L French oak chips), and 560 mg/L (5 g/L French oak chips). Increased alcohol amounts during ageing can be caused by acid-catalyzed ester hydrolysis, as reported by Rapp et al. [22]. The type of oak chips did not significantly influence methanol, propanol, 2-methyl-1-butanol, and 2-phenylethanol production. In the minor group, hexanol was the highest alcohol, and its concentration was significantly dependent on ageing time and type and dosages of oak chips. This compound is recognized as a “leafy” alcohol and has a “grassy” note [23]. Small changes were observed in the content of furfuryl alcohol, 2-phenylethanol, and benzyl alcohol during the ageing process (Figure 1). Furfuryl alcohol is considered to originate from the reduction of furfural, after alcoholic and malolactic fermentation, during wine ageing with oak chips [5,24]. Moreover, furfuryl alcohol transformation depends on enzymatic or microbiological activities, as well as on oenological parameters [25].

#### 3.2.2. Carbonyls

In the carbonyl group, two major and five minor aroma compounds were determined, respectively, acetaldehyde and acetoin and heptanal, octanal, furfural, benzaldehyde, and 5-methylfurfural. Volatile aldehydes constitute a broad group of chemical compounds subjected to concentration alterations during the ageing of wines. Aroma changes due to oxidation processes is frequently linked to the synthesis of aldehydes [26]. Acetaldehyde is an aroma compound that modifies wine’s aroma as a result of prolonged wine contact with oxygen [27,28]. Moreover, acetaldehyde is generated by yeast metabolism, and its presence is correlated with fruity notes and nuts or dried fruits aromas. Sulphur dioxide impedes this aldehyde reaction, but in some cases, even in the presence of high amounts of SO_2_, some acetaldehyde can form [27]. Acetoin is produced during fermentation by the microbial activity of lactic acid bacteria and yeasts. Our results showed similar variation trends in the concentrations of acetaldehyde and acetoin, as presented in Table 2. Moreover, acetaldehyde and acetoin concentrations increased significantly in wines treated with both types of oak chips and were significantly dependent on all three factors (time, and oak chips’ type and dosage).

Furan is generally obtained through the Maillard reaction, consisting in the decomposition of carbohydrates under high temperature and dehydration. These reactions take place in the course of fermentation and ageing, and furan is often considered an ageing marker [29]. The compound 5-methylfurfural derives from hexoses present in cellulose, and furfural originates from pentoses, the main constituents of hemicelluloses. In wines aged with American oak chips for 1.5 months, the levels of furfural were 208 µg/L (3 g/L) and 518 µg/L (5 g/L), whereas in wines aged with French oak chips, they were 904 µg/L (3 g/L) and 957 µg/L (5 g/L). After 3 months of ageing, the concentrations of furfural were 534 µg/L (3 g/L) and 433 µg/L (5 g/L) for American oak chip-treated wines and 1336 µg/L (3 g/L) and 865 µg/L (5 g/L) for French oak chip-treated wines. Generally, volatile aldehydes compounds increase during ageing, but the role that aldehydes play in the overall aroma of red wine is poorly understood [30]. According to our results, French oak chips contain higher levels of furanic aldehydes in comparison with American oak chips, with furfural and 5-methylfurfural being the most abundant (Figure 1). Octanal, furfural, benzaldehyde, and 5-methylfurfural appearance was significantly dependent on ageing time and type and dosages of oak chips. Heptanal appearance was dependent only on the ageing time and dosage factors.

#### 3.2.3. Carboxylic Acids

Fatty acids are produced by yeast and bacteria biosynthesis during the fermentation step in winemaking [31]. Acids could impact the sensory characteristics and have been described as imparting fruity, cheese, fatty, and rancid notes [32]. Four acids were detected in our aged wines: butanoic acid, octanoic acid, decanoic acid, and hexanoic acid. As shown in Table 2, octanoic acid was the most abundant compound of this group, with concentrations in the American oak chip-treated samples of 3264 µg/L (3 g/L) and 3765 µg/L (5 g/L) and in the French oak chip-treated samples of 3930 µg/L (3 g/L) and 3867 µg/L (5 g/L) at 1.5 months. After 3 months of ageing, the concentration in American oak chip-treated samples were 7218 µg/L (3 g/L) and 5434 µg/L (5 g/L), while in French oak chip-treated samples, they were 6671 µg/L (3 g/L) and 5727 µg/L (5 g/L). The concentrations of all carboxylic acids were significantly dependent on the ageing time and type and dosage of oak chips. One exception was octanoic acid, whose occurrence was not significantly influenced by any factor. As for butanoic acid, a slight decrease in its concentration was observed during the ageing process of wine samples (Figure 1). In contrast, the contents of octanoic acid, decanoic acid, and hexanoic acid in wines aged for 3 months were found to be higher than those in wines aged for 1.5 months, for both American and French oak chip-treated samples. The decrease in acids concentration could be provoked by the loss of volatile acids and esterification reactions between alcohols and acids during the ageing process [33].

#### 3.2.4. Esters

Esters are principally synthesized by yeasts during the fermentation stage or appear after fatty acid esterification with ethanol and acetic acid through the fermentation and ageing processes. They are considered highly positive flavor attributes of young wine’s aroma, contributing to its fruity, floral, and sweet notes [34]. During the ageing process, low temperatures are required, because high temperatures may impair ester formation by causing volatilization and hydrolysis. Esters are generally produced in the final part of the fermentation step. They gradually hydrolyze during the ageing process until wine’s chemical equilibrium is achieved. These modifications in wine esters composition are influenced by ageing temperature and pH, as well as by the alcoholic degree of the final products [35].

We identified in total 17 esters, of which 3 major and 14 minor esters (Table 2). In the first group, ethyl lactate was the most prominent representative, with concentrations for American oak chip-treated samples of 126 mg/L (3 g/L) and 223 mg/L (5 g/L) and for French oak chip-treated samples of 92 mg/L (3 g/L) and 193 mg/L (5 g/L) at 1.5 months of ageing. Upon ageing for 3 months, the concentration of ethyl lactate increased in American oak chip-treated samples to 241 mg/L (3 g/L) and 243 mg/L (5 g/L) and in French oak chip-treated samples to 252 mg/L (3 g/L) and 259 mg/L (5 g/L). It was observed that the concentration of ethyl lactate fluctuated slightly, even if no significant difference was observed between wines treated with the two types of oak chips. Ethyl lactate is mainly formed by lactic bacteria and considerably contributes to sustaining wine aroma characteristics. The concentrations of ethyl acetate, ethyl lactate, and diethyl succinate were found to be dependent on ageing time and oak chips’ type and dosage.

Regarding the second group of esters, isoamyl acetate presented the highest concentrations in American oak chip-treated samples, corresponding to 1146 µg/L (3 g/L) and 1417 µg/L (5 g/L), and in French oak chip-treated samples of 1400 µg/L (3 g/L) and 1891 µg/L (5 g/L) after 1.5 months of ageing. After 3 months of ageing, the concentrations in American oak chip-treated samples increased to 2806 µg/L (3 g/L) and 2213 µg/L (5 g/L), and those in French oak chip-treated samples to 2270 µg/L (3 g/L) and 2084 µg/L (5 g/L). The increased values of isoamyl acetate could be caused by its release from the residual lees, due to the lack of filtration of the wines at the sampling stage. On the other hand, isoamyl acetate is considered to be produced by a high rate of esterification reactions between isoamyl alcohol and acetic acid. The appearance of this compound depended only on the ageing time. Isoamyl acetate is used in the food industry because of its characteristic banana aroma [36]. Also, ethyl octanoate, which smells of brandy, has an important role in wine aroma.

The concentrations of ethyl propionate, ethyl butanoate, ethyl octanoate, ethyl 2-methyloctanoate, phenylethyl acetate, and ethyl decanoate showed similar variation trends during the ageing process. This increase of fruity volatiles in red wines aged for 3 months in the presence of oak chips with regard to wines aged for 1.5 months has been previously suggested for ethyl butanoate, ethyl hexanoate [37], and isoamyl acetate [38]. This may be caused by esters synthesis during ageing, due to their levels being below the equilibrium concentration at fermentation, and by their release into wine during yeast cellular lysis [38]. On the other hand, ethyl isobutanoate, ethyl furoate, ethyl dodecanoate, ethyl tetradecanoate, ethyl hexadecanoate, and hexyl hexanoate decreased slightly after 3 months of ageing (Figure 1). Diaz-Maroto et al. [34] suggested that the quantities of ethyl esters derived from the lipid metabolism of yeast decrease during winemaking and especially during ageing.

#### 3.2.5. Lactones

Lactones are important aroma compounds in red wines. In this study, we identified four lactones: crotonolactone, butyrolactone, nonalactone, and decalactone. Butyrolactone was the most abundant compound of this group, with concentration in American oak chip-treated samples of 411 µg/L (3 g/L) and 299 µg/L (5 g/L) and in French oak chip-treated samples of 469 µg/L (3 g/L) and 815 µg/L (5 g/L) at 1.5 months. After 3 months of ageing, the concentration of this compound increased in American oak chip-treated samples to 802 µg/L (3 g/L) and 816 µg/L (5 g/L), whereas it decreased in French oak chip-treated samples to 360 µg/L (3 g/L) and 658 µg/L (5 g/L). Decalactone concentration was significantly dependent on all three factors (Table 2). Moreover, crotonolactone, butyrolactone, and nonalactone concentrations were found to be dependent on ageing time and oak chip dosage. Nonalactone is considered important for the flavor of aged red wine due to its powerful odor of prune [39].

#### 3.2.6. Terpenes

In the terpene group, only limonene was identified in aged wines, and its concentration was significantly dependent on ageing time and oak chips’ type and dosage.

#### 3.2.7. Volatile Phenols

Guaiacol and 4-vinylguaiacol were identified in samples treated with American and French oak chips. In samples treated with American oak chips, 4-vinylguaiacol their concentrations were 21 µg/L (3 g/L) and 27 µg/L (5 g/L), and in samples treated with French oak chips, they were 23 µg/L (3 g/L) and 51 µg/L (5 g/L) at 1.5 months of ageing. The concentrations after 3 months of ageing with American oak chips were 70 µg/L (3 g/L) and 51 µg/L (5 g/L), and the correspondent ones in the presence of French oak chips were 95 µg/L (3 g/L) and 35 µg/L (5 g/L). The concentrations of both volatile phenols were significantly dependent on the ageing time and oak chips’ type and dosage. Guaiacol and 4-vinylguaiacol have smoky aromas, and their concentrations are indicators of the relative toast level of the oak chips, since they form almost exclusively by the degradation of lignin during the toasting process. Also, 4-vinylguaiacol and 4-vinylphenol can be produced by enzymatic or thermal decarboxylation from cinnamic acids [40].

#### 3.2.8. Oak Compounds

Oak lactones are major volatile compounds present in oak wood and are considered the most important aroma contributors of ageing wines, especially the *cis* isomer and the *trans* isomer [41]. Moreover, they are markers of oak wood ageing process and crucial contributors to the woody flavors of aged wines. *Cis*-whiskey lactone was the most abundant component of this group, with concentration in American oak chip-treated samples of 190 µg/L (3 g/L) and 416 µg/L (5 g/L) and in French oak chip-treated samples of 93 µg/L (3 g/L) and 100 µg/L (5 g/L) after 1.5 months of ageing. After 3 months of ageing, its concentration increased in American oak chip-treated samples to 430 µg/L (3 g/L) and 618 µg/L (5 g/L) and in French oak chip-treated samples to 143 µg/L (3 g/L) and 172 µg/L (5 g/L) (Table 2 and Figure 1). The concentrations of oak compounds were significantly dependent on all three factors (ageing and oak chips’ time and dosage). Oak lactones in wines are the outcome of oak wood extraction processes during ageing and are a function of oak wood type and doses [42,43]. The ratio of *cis/trans* isomers has been frequently used in winemaking as a distinguishing marker of aged wines, with values close to 2 for French oak wood and higher than 5 for American oak wood [42,44]. However, there are a many other factors which may affect this ratio, especially the degree of toasting, oak age, duration of the contact with wine, and type of wood [45]. In this study, we observed that the *cis/trans* lactone ratio was around 3.5 in American oak chip-treated samples and 1 in French oak chip-treated samples.

### 3.3. Box Plot and Principal Component Analysis

Among all the 47 aroma compounds identified in wines aged with American and French oak chips, particular attention was given to furfural, 5-methylfurfural, guaiacol, 4-vinylguaiacol, trans-whiskey lactone, and *cis*-whiskey lactone due to their potential positive impact on wine aroma properties and because they are specific compounds released from oak wood. Therefore, a box plot was realized with these six key compounds, by using their cumulative concentrations for both periods of ageing, in order to distinguish between the two oak species and dosages used.

**Figure 2:**
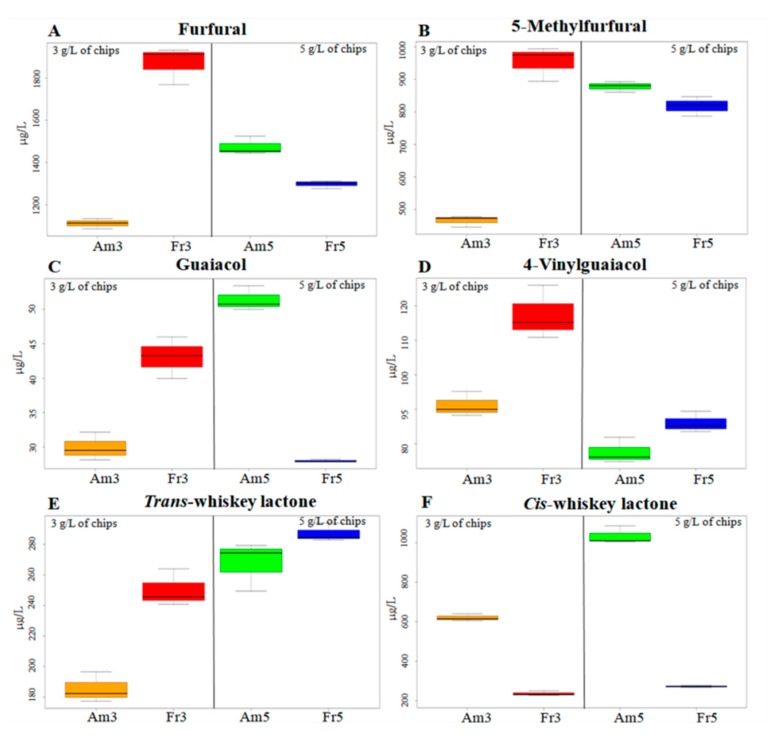
Box plot of key compounds of wine aged with different types of oak chips.

Furfural had the highest median and values in wines treated with French oak chips compared to those treated with American oak chips in the case of the treatment with 3 g/L of chips, while it presented the lowest median for the treatment with 5 g/L oak chips. The compound 5-methylfurfural presented the same behavior as furfural. Thus, both compounds increased in samples treated with French oak chips at 3 g/L and decreased when used 5 g/L of chips was used. In wines treated with American oak chips, these compounds showed a different trend when compared with those observed for wines treated with French oak chips (Figure 2A,B). As previously found [46], furfural and 5-methylfurfural levels were higher in French oak chip-treated samples also in or study.

Our results indicated that guaiacol had the largest distribution range as well as the highest median and values in wines treated with French oak chips as compared to those treated with American oak chips at 3 g/L. In contrast, for the other dosage used, the behavior was exactly the opposite: the highest median was found for American oak chip-treated samples, and the lowest median was found for French oak chip-treated samples. Concerning 4-vinylguaiacol, the highest median and values were revealed in samples treated with French oak chips at both dosages, as compared with American oak chip-treated samples (Figure 2C,D).

For *trans*-whiskey lactone, the highest value and median were found in wines treated with French oak chips at both dosages (Figure 2E,F). In contrast, for *cis*-whiskey lactone, the distribution range and median were noticeably higher in American oak chip-treated samples than in French oak chip-treated samples, at dosages of 3 g/L and 5 g/L. These findings are in accordance with those of previous investigations, which demonstrated that American oak chips (*Q. alba*) contains significantly higher amounts of *cis*-whiskey lactone as compared with French oak chips [47].

**Figure 3:**
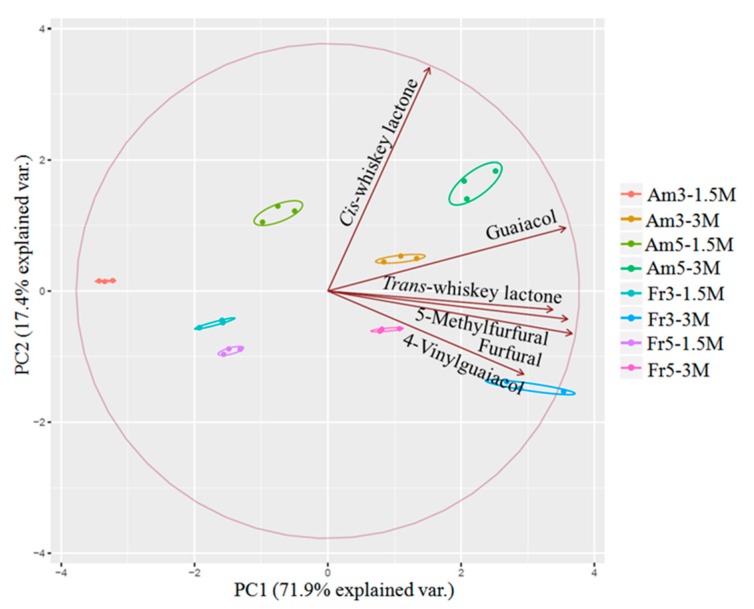
Principal component analysis of wines aged with American and French oak chips. PC1: first principal component; PC2: second principal component.

PCA is one of the multivariate statistical analysis methods that are used intensely in the food and beverage area and is useful to decrease data dimensionality, while keeping patterns. In this research, PCA (Figure 3) was used to examine aged wines with two types of oak chips (American and French), two dosages of oak chips (3 g/L and 5 g/L), and two different ageing times (1.5 and 3 months). Furthermore, PCA was used to investigate the contribution of specific aroma compounds to each principal component. As it can be observed from Figure 3, the first principal component (PC1), accounting for 71.9% of the total variance, relates to the increase in compound intensity with increased ageing time. PC1 led to the separation of the samples treated with American and French oak chips between those aged for 1.5 months and those aged 3 months, since the samples aged for 3 months in the presence of American and French oak chips were grouped on the positive side of PC1, while the samples aged for 1.5 months were located on the negative side of PC1. The second principal component (PC2) accounted for 17.4% of the variation, separating samples high in *cis*-whiskey lactone from those high in 4-vinylguaiacol and furfural. Furthermore, PC2 contributed to the differentiation of wines aged with American oak chips from those aged with French oak chips. Samples aged for 1.5 months with French oak chips were located on the negative side of PC2, whereas the American oak chip-treated samples were located on the positive side. The principal component analysis shows that the French oak chips aged for 1.5 months have similar wine flavor profiles; in contrast, clear differences were observed when the wines were aged for 3 months.

### 3.4. Sensorial Analysis

Ageing is considered the winemaking process that leads to profound chemical and aroma changes, due to the extraction of aromatic compounds from oak wood. The process results in richness and increased complexity of flavor characteristics.

The volatile compounds extracted from oak wood chips are described as important olfactory factors, generating notes of coconut, wood, vanilla, caramel, and spices [16,48]. Generally, these compounds have low detection thresholds and may be identified by specialized tasters in aged wine at very low concentrations.

**Figure 4:**
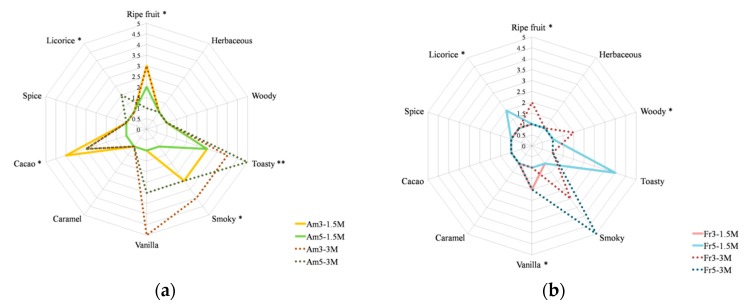
Sensory analysis of American (**a**) and French (**b**) oak chip-treated wines aged for 1.5 and 3 months.

In order to better visualize the variation of the aromatic profiles during the ageing process, a radar map of red wines aged for different times and in the presence of different oak chips’ dosages and types was generated using sensorial analysis done by an expert tasting panel. As shown in Figure 4A,B, the woody, toasty, smoky, and vanilla sensations were perceived as more intense in wines treated with oak chips for 3 months than in those treated for 1.5 months. These findings are in accordance with the presence in wines of oak lactones and volatile phenols, the main responsible compounds for these attributes. Furthermore, it is important to notice that in wines aged with American oak chips, vanilla and cacao notes were much more pronounced than in wines aged with French oak chips. Moreover, the treatment with oak chips produced important sensory changes in wines and drastically reduced the olfactory characteristics of Fetească neagră wine. Wines samples presented more intense smoke and toasty notes, as an effect of increased amounts of volatile phenols and furan derivatives, similar to what observed by Chatonnet and Dubourdieu [49].

## 4. Conclusions

The wines’ chemical composition and sensory properties varied significantly in relation to the type of chips used for ageing. French oak chip-treated samples presented higher concentrations of ageing compounds (furfural, 5-methylfurfural, guaiacol, 4-vinylguaiacol, and *trans*-whiskey lactone) when 3 g/L of oak chips was used. For samples aged with 5 g/L of oak chips, the concentration decreased. Meanwhile, American oak chips samples contained higher concentrations of *cis*-whiskey lactone in wines treated with both dosages as compared with wines treated with French oak chips. Considering all results, it can be concluded that ageing with American and French oak chips is an efficient alternative method that could enhance the sensory complexity of wines. Wines aged with chips of French and American oak could be distinguished due to the content of six key volatile compounds. Ageing with oak chips causes the degradation of flavor compounds associated with fruity notes but leads to the acquisition of new oak-related aromas. Nevertheless, these changes improve wine quality and are consistent with consumers’ preferences.

## Figures and Tables

**Table 1:** Conventional analyses of red wines aged with oak chips. Am3: 3 g/L American oak chips, Am5: 5 g/L American oak chips, Fr3: 3 g/L French oak chips, Fr5: 5 g/L French oak chips for 1.5 and 3 months; multivariate analysis of variance (MANOVA) taking as factors time (1.5 and 3 months), types of oak chips (American and French,) and dosage (3 and 5 g/L); total polyphenols index (TPI) at 280 nm; absorbance A.

Compounds	American Oak				French Oak				MANOVA		
	1.5 Months		3 Months		1.5 Months		3 Months		Time	Type	Dosage
	Am3	Am5	Am3	Am5	Fr3	Fr5	Fr3	Fr5			
Ethanol (%*v*/*v*)	14.96 ± 0.09 ^a^	14.96 ± 0.14 ^a^	14.94 ± 0.03 ^a^	14.94 ± 0.02 ^a^	14.96 ± 0.01 ^a^	14.96 ± 0.12 ^a^	14.94 ± 0.01 ^a^	14.94 ± 0.05 ^a^	**	ns	ns
Reducing sugars (g/L)	2.36 ± 0.01 ^ab^	2.37 ± 0.02 ^b^	2.36 ± 0.01 ^ab^	2.36 ± 0.04 ^ab^	2.35 ± 0.01 ^a^	2.36 ± 0.05 ^ab^	2.35 ± 0.01 ^a^	2.35 ± 0.05 ^a^	ns	ns	ns
Volatile acidity (acetic acid g/L)	0.52 ± 0.01 ^a^	0.52 ± 0.01 ^a^	0.84 ± 0.01 ^d^	0.84 ± 0.01^d^	0.52 ± 0.01 ^a^	0.52 ± 0.01 ^a^	0.69 ± 0.02 ^b^	0.81 ± 0.00 ^c^	***	***	***
Total acidity (tartaric acid g/L)	5.91 ± 0.05 ^c^	5.89 ± 0.00 ^b^	5.86 ± 0.00 ^a^	5.85 ± 0.04 ^a^	5.92 ± 0.08 ^c^	5.91 ± 0.04 ^c^	5.89 ± 0.00 ^b^	5.85 ± 0.02 ^a^	***	**	***
pH	3.66 ± 0.01 ^a^	3.66 ± 0.01 ^a^	3.66 ± 0.01 ^a^	3.66 ± 0.01 ^a^	3.66 ± 0.02 ^a^	3.66 ± 0.01 ^a^	3.66 ± 0.02 ^a^	3.66 ± 0.01 ^a^	ns	ns	ns
Free SO_2_ (mg/L)	49.82 ± 1.6 ^e^	50.01 ± 1.6 ^f^	38.52 ± 1.1 ^a^	42.11 ± 1.3 ^b^	51.75 ± 2.2 ^g^	51.95 ± 1.3 ^h^	43.42 ± 1.4 ^c^	45.14 ± 1.2 ^d^	***	***	***
Total SO_2_ (mg/L)	149.73 ± 3.7 ^g^	135.42 ± 3.5 ^b^	134.02 ± 2.7 ^a^	142.85 ± 2.8 ^e^	149.98 ± 2.9 ^h^	147.05 ± 2.3 ^f^	136.74 ± 2.8 ^c^	142.16 ± 2.4 ^d^	***	***	***
TPI (AU 280)	3.23 ± 0.05 ^bc^	3.16 ± 0.09 ^b^	2.90 ± 0.03 ^a^	3.00 ± 0.09 ^a^	3.42 ± 0.07 ^d^	3.35 ± 0.03 ^cd^	3.20 ± 0.1 ^b^	3.27 ± 0.08 ^bc^	***	***	ns
L *	30.5 ± 0.9 ^c^	28.4 ± 0.9 ^b^	28.7 ± 0.9 ^b^	25.2 ± 0.8 ^a^	28.2 ± 0.8 ^b^	25.9 ± 0.8 ^a^	26.4 ± 0.8 ^a^	25.6 ± 0.8 ^a^	***	***	***
a *	54 ± 2 ^ab^	54 ± 2 ^ab^	55 ± 2 ^b^	52 ± 2 ^a^	54 ± 2 ^ab^	53 ± 2 ^ab^	53 ± 2 ^ab^	52 ± 2 ^a^	ns	ns	*
b *	32 ± 1 ^a^	34 ± 1 ^ab^	38 ± 1^c^	36 ± 1 ^bc^	34 ± 1 ^ab^	36 ± 1 ^c^	36 ± 1 ^bc^	37 ± 1 ^c^	***	ns	**
A_420_	1.869 ± 0.01 ^a^	2.077 ± 0.01 ^c^	2.117 ± 0.01 ^d^	2.369 ± 0.02 ^g^	2.038 ± 0.01 ^b^	2.318 ± 0.02 ^f^	2.209 ± 0.02 ^e^	2.373 ± 0.02 ^h^	***	***	***
A_520_	0.127 ± 0.02 ^c^	0.122 ± 0.02 ^b^	0.112 ± 0.02 ^b^	0.107 ± 0.02 ^a^	0.135 ± 0.02 ^d^	0.135 ± 0.02 ^d^	0.110 ± 0.01 ^a^	0.108 ± 0.01 ^a^	***	***	**
A_620_	0.524 ± 0.03 ^a^	0.569 ± 0.02 ^c^	0.560 ± 0.03 ^b^	0.659 ± 0.04 ^g^	0.572 ± 0.03 ^c^	0.634 ± 0.04 ^e^	0.622 ± 0.04 ^d^	0.646 ± 0.04 ^f^	***	***	***

*: *p* ≤ 0.05; **: *p* ≤ 0.01; ***: *p* ≤ 0.001; ns: not significant; different letters (^a, b, c, d, e, h, g^) show significant differences at 95% confidence level.

**Table 2:** Quantitative analysis of aroma compounds of red wines aged with oak chips. All values are expressed as means (mg/L and µg/L) ± standard deviation (SD). Multivariate analysis of variance (MANOVA) taking as factors time (1.5 and 3 months), types (American and French), and dosage (3 and 5 g/L) of oak chips.

Compounds	American Oak				French Oak				MANOVA		
	1.5 Months		3 Months		1.5 Months		3 Months		Time	Type	Dosage
	Am3	Am5	Am3	Am5	Fr3	Fr5	Fr3	Fr5			
**Major alcohols**	898 ± 5 ^a^	914 ± 7 ^ab^	939 ± 22 ^c^	968 ± 9 ^d^	912 ± 6 ^ab^	916 ± 12 ^ab^	920 ± 2 ^bc^	958 ± 12 ^d^	***	ns	***
*Subtotal (%)*	*74.77*	*68.36*	*66.69*	*66.85*	*77.55*	*68.87*	*65.06*	*64.30*			
Methanol (mg/L)	221 ± 3 ^a^	226 ± 4 ^a^	236 ± 10 ^bc^	241 ± 4 ^c^	228 ± 2 ^ab^	229 ± 4 ^ab^	229 ± 1 ^ab^	245 ± 8 ^c^	***	ns	**
Propanol (mg/L)	36 ± 0.2 ^a^	37 ± 0.3 ^a^	38 ± 2 ^bc^	40 ± 1 ^c^	36 ± 1 ^a^	37 ± 1 ^a^	37 ± 0.4 ^ab^	38 ± 1 ^bc^	***	ns	*
Isobutanol (mg/L)	48 ± 1 ^ab^	49 ± 1^bc^	49 ± 2 ^bc^	50 ± 0.4 ^c^	47 ± 2 ^a^	47 ± 1 ^a^	47 ± 0.3 ^a^	49 ± 1 ^bc^	ns	**	*
2-methyl-1-butanol (mg/L)	531 ± 7 ^a^	534 ± 2 ^a^	553 ± 11 ^b^	571 ± 1 ^c^	537 ± 4 ^a^	538 ± 8 ^a^	539 ± 4 ^a^	560 ± 2 ^b^	***	ns	**
2-phenylethanol (mg/L)	62 ± 1 ^a^	67 ± 1 ^b^	62 ± 2 ^a^	68 ± 4 ^b^	64 ± 3 ^ab^	66 ± 4 ^ab^	68 ± 3 ^b^	69 ± 4 ^b^	ns	ns	**
**Minor alcohols**	1643 ± 67 ^a^	2296 ± 76 ^b^	5241 ± 65 ^e^	3055 ± 77 ^c^	2360 ± 74 ^b^	1509 ± 62 ^a^	3442 ± 95 ^d^	2590 ± 86 ^b^	***	***	***
*Subtotal (%)*	*18.50*	*19.98*	*23.57*	*17.46*	*20.05*	*11.27*	*17.79*	*14.45*			
Hexanol (µg/L)	1201 ± 81 ^a^	1668 ± 112 ^b^	4091 ± 116 ^e^	2362 ± 173 ^d^	1722 ± 75 ^bc^	1063 ± 55 ^a^	2370 ± 75 ^d^	1853 ± 102 ^c^	***	***	***
E-3-hexenol (µg/L)	102 ± 6 ^a^	149 ± 5 ^c^	193 ± 9 ^d^	148 ± 11 ^c^	130 ± 7 ^b^	109 ± 3 ^a^	237 ± 13 ^e^	147 ± 9 ^c^	***	*	***
E-2-hexenol (µg/L)	261 ± 27 ^ab^	409 ± 15 ^bc^	965 ± 31 ^e^	458 ± 24 ^c^	441 ± 11 ^c^	251 ± 11 ^a^	751 ± 16 ^d^	481 ± 17 ^c^	***	ns	***
Furfuryl alcohol (µg/L)	72 ± 4 ^de^	63 ± 6 ^bc^	76 ± 2 ^ef^	68 ± 2 ^cd^	59 ± 4 ^b^	78 ± 7 ^ef^	50 ± 2 ^a^	80 ± 1 ^f^	ns	ns	***
Benzyl alcohol (µg/L)	8 ± 0.5 ^a^	7 ± 0.1 ^a^	16 ± 1.5 ^b^	20 ± 1 ^c^	7 ± 0.2 ^a^	7 ± 0.1 ^a^	35 ± 1 ^e^	29 ± 1 ^d^	***	***	ns
**Major carbonyls**	124 ± 4 ^a^	140 ± 4 ^b^	164 ± 4 ^cd^	169 ± 6 ^d^	116 ± 5 ^a^	159 ± 7 ^c^	181 ± 7 ^e^	202 ± 5 ^f^	***	***	***
*Subtotal (%)*	*10.32*	*10.47*	*11.65*	*11.67*	*9.86*	*11.95*	*12.80*	*13.56*			
Acetaldehyde (mg/L)	51 ± 1 ^b^	56 ± 2 ^c^	57 ± 1 ^cd^	60 ± 1 ^cd^	47 ± 2 ^a^	61 ± 4 ^d^	69 ± 3 ^e^	70 ± 3 ^e^	***	***	***
Acetoin (mg/L)	73 ± 3 ^a^	84 ± 4 ^b^	107 ± 5 ^d^	109 ± 5 ^d^	69 ± 2 ^a^	98 ± 4 ^c^	112 ± 4 ^d^	132 ± 7 ^e^	***	***	***
**Minor carbonyls**	401 ± 16 ^a^	894 ± 43 ^bc^	1435 ± 38 ^d^	1727 ± 53 ^f^	950 ± 41 ^c^	820 ± 37 ^b^	2153 ± 52 ^g^	1563 ± 33 ^e^	***	***	ns
*Subtotal (%)*	*4.52*	*7.78*	*6.45*	*9.87*	*8.07*	*6.12*	*11.13*	*8.72*			
Heptanal (µg/L)	66 ± 2 ^de^	54 ± 2 ^a^	61 ± 3 ^c^	63 ± 3 ^cd^	56 ± 1 ^ab^	66 ± 1 ^de^	59 ± 2 ^bc^	67 ± 3 ^e^	*	ns	*
Octanal (µg/L)	23 ± 1^c^	5 ± 0.3 ^a^	5 ± 0.2 ^a^	15 ± 1 ^b^	26 ± 2 ^d^	23 ± 2 ^c^	13 ± 1 ^b^	31 ± 3 ^e^	***	***	*
Furfural (µg/L)	208 ± 12 ^a^	518 ± 25 ^c^	904 ± 23 ^de^	957 ± 50 ^e^	534 ± 41 ^c^	433 ± 38 ^b^	1336 ± 50 ^f^	865 ± 20 ^d^	***	***	**
Benzaldehyde (µg/L)	17 ± 1.5 ^a^	39 ± 2 ^c^	86 ± 4 ^e^	92 ± 3 ^f^	36 ± 3 ^c^	29 ± 3 ^b^	88 ± 5 ^ef^	52 ± 3 ^d^	***	***	**
5-methylfurfural (µg/L)	87 ± 4 ^a^	278 ± 18 ^b^	379 ± 17^c^	601 ± 22 ^e^	297 ± 21 ^b^	270 ± 8 ^b^	657 ± 32 ^f^	548 ± 22 ^d^	***	***	***
**Carboxylic acids**	3718 ± 86 ^a^	4304 ± 57 ^b^	8037 ± 75 ^f^	6137 ± 76 ^d^	4541 ± 69 ^b^	5161 ± 82 ^c^	7728 ± 95 ^f^	6783 ± 76 ^e^	***	***	***
*Subtotal (%)*	*41.87*	*37.46*	*36.15*	*35.07*	*38.58*	*38.53*	*39.94*	*37.86*			
Butanoic acid (µg/L)	40 ± 3 ^de^	46 ± 2 ^f^	27 ± 2 ^b^	19 ± 1 ^a^	43 ± 3 ^ef^	37 ± 2 ^d^	60 ± 4 ^g^	23 ± 2 ^b^	***	***	***
Octanoic acid (µg/L)	3264 ± 251 ^a^	3765 ± 129 ^b^	7218 ± 256 ^e^	5434 ± 209 ^c^	3930 ± 97 ^b^	3867 ± 146 ^b^	6671 ± 170 ^d^	5727 ± 197 ^c^	***	ns	***
Decanoic acid (µg/L)	401 ± 33 ^a^	481 ± 18 ^b^	749 ± 21 ^d^	652 ± 28 ^c^	526 ± 31 ^b^	1248 ± 50 ^g^	927 ± 28 ^e^	988 ± 51 ^f^	***	***	***
Hexanoic acid (µg/L)	13 ± 0.4 ^a^	12 ± 1 ^a^	44 ± 5 ^c^	32 ± 2 ^b^	42 ± 2 ^c^	9 ± 0.5 ^a^	69 ± 3 ^d^	46 ± 3 ^c^	***	***	***
**Major esters**	179 ± 9 ^b^	283 ± 5 ^d^	305 ± 17 ^e^	311 ± 5 ^e^	148 ± 6 ^a^	255 ± 11 ^c^	313 ± 9 ^e^	330 ± 9 ^f^	***	ns	***
*Subtotal (%)*	*14.90*	*21.17*	*21.66*	*21.48*	*12.59*	*19.17*	*22.14*	*22.15*			
Ethyl acetate (mg/L)	32 ± 0.2 ^ab^	32 ± 0.5 ^ab^	32 ± 1 ^ab^	35 ± 1 ^bc^	30 ± 0.4 ^a^	31 ± 2 ^ab^	31 ± 0.4 ^ab^	34 ± 2 ^c^	**	*	***
Ethyl lactate (mg/L)	126 ± 7 ^b^	223 ± 6 ^d^	241 ± 15^e^	243 ± 3 ^e^	92 ± 6 ^a^	193 ± 7 ^c^	252 ± 8 ^ef^	259 ± 7 ^f^	***	*	***
Diethyl succinate (mg/L)	21 ± 2 ^a^	27 ± 2 ^bc^	32 ± 1 ^de^	33 ± 1 ^e^	26 ± 1 ^b^	30 ± 2 ^cd^	30 ± 1 ^d^	37 ± 1 ^f^	***	**	***
**Minor esters**	2276 ± 46 ^a^	2834 ± 44 ^b^	5736 ± 62^d^	4596 ± 64 ^c^	2985 ± 59 ^b^	4623 ± 53 ^c^	4833 ± 65 ^c^	5705 ± 83 ^d^	***	***	***
*Subtotal (%)*	*25.63*	*24.67*	*25.80*	*26.27*	*25.36*	*34.52*	*24.98*	*31.84*			
Ethyl propionate (µg/L)	221 ± 10 ^a^	320 ± 20 ^b^	585 ± 30 ^d^	576 ± 25 ^d^	332 ± 20 ^b^	193 ± 8 ^a^	574 ± 42 ^d^	408 ± 26 ^c^	***	***	***
Ethyl isobutanoate (µg/L)	5 ± 0.3 ^b^	30 ± 1.5 ^e^	11 ± 1 ^c^	9 ± 0.4 ^c^	55 ± 4 ^f^	2 ± 0.2 ^a^	16 ± 1 ^d^	8 ± 0.3 ^c^	***	***	***
Ethyl butanoate (µg/L)	253 ± 14 ^a^	330 ± 5 ^b^	565 ± 18 ^d^	555 ± 27 ^d^	354 ± 26 ^b^	355 ± 19 ^b^	558 ± 22 ^d^	410 ± 12 ^c^	***	ns	*
Isoamylacetate (µg/L)	1146 ± 76 ^a^	1417 ± 35 ^b^	2806 ± 117 ^e^	2213 ± 172 ^d^	1400 ± 75 ^b^	1891 ± 143 ^c^	2270 ± 187 ^d^	2084 ± 106 ^cd^	***	ns	ns
Ethyl furoate (µg/L)	7 ± 0.5 ^e^	5 ± 0.4 ^c^	5 ± 0.2 ^c^	3 ± 0.1 ^b^	3 ± 0.4 ^b^	6 ± 0.5 ^d^	3 ± 0.1 ^ab^	2 ± 0.1 ^a^	***	***	*
Ethyl octanoate (µg/L)	332 ± 22 ^a^	464 ± 11 ^b^	1288 ± 72 ^e^	880 ± 26 ^c^	502 ± 34 ^b^	1789 ± 85 ^f^	991 ± 7 ^d^	2216 ± 126 ^g^	***	***	***
Ethyl 2-methyloctanoate (µg/L)	9 ± 1 ^a^	9 ± 0.6 ^a^	22 ± 1 ^d^	16 ± 1 ^c^	12 ± 0.4 ^b^	10 ± 0.1 ^a^	15 ± 1 ^c^	15 ± 1 ^c^	***	**	***
Phenylethyl acetate (µg/L)	58 ± 3 ^a^	76 ± 5 ^b^	126 ± 8 ^d^	109 ± 6 ^c^	76 ± 5 ^b^	105 ± 5 ^c^	122 ± 7 ^d^	100 ± 5 ^c^	***	**	ns
Ethyl decanoate (µg/L)	93 ± 6 ^b^	77 ± 2 ^a^	146 ± 9 ^d^	107 ± 3 ^c^	114 ± 4 ^c^	169 ± 9 ^e^	157 ± 4 ^d^	284 ± 9 ^f^	***	***	***
Ethyl vanillate (µg/L)	8 ± 0.4 ^a^	9 ± 0.1 ^b^	10 ± 0.8 ^c^	9 ± 0.5 ^b^	8 ± 0.2 ^a^	8 ± 0.2 ^a^	10 ± 0.2 ^c^	9 ± 0.2 ^b^	***	ns	ns
Ethyl dodecanoate (µg/L)	101 ± 6 ^c^	54 ± 2 ^a^	135 ± 10 ^d^	72 ± 4 ^b^	92 ± 5 ^c^	55 ± 2 ^a^	76 ± 4 ^b^	135 ± 9 ^d^	***	ns	***
Ethyl tetradecanoate (µg/L)	23 ± 1b ^c^	23 ± 1 ^bc^	22 ± 2 ^bc^	25 ± 1 ^c^	21 ± 1 ^ab^	23 ± 1 ^bc^	23 ± 1 ^bc^	19 ± 1 ^a^	ns	**	ns
Ethyl hexadecanoate (µg/L)	17 ± 1 ^d^	18 ± 1 ^d^	14 ± 1 ^b^	22 ± 1 ^e^	15 ± 1 ^c^	17 ± 1 ^d^	18 ± 1 ^d^	12 ± 1 ^a^	ns	***	***
Hexyl hexanoate (µg/L)	1.5 ± 0.1 ^e^	1 ± 0.1 ^d^	0.5 ± 0.1 ^b^	0.7 ± 0.1 ^c^	1 ± 0.1 ^d^	0.7 ± 0.1 ^c^	0.4 ± 0.1 ^a^	0.5 ± 0.1 ^b^	***	***	*
**Lactones**	565 ± 31 ^a^	575 ± 14 ^a^	1120 ± 23 ^f^	1115 ± 21 ^f^	703 ± 13 ^b^	1000 ± 34 ^e^	752 ± 23 ^c^	860 ± 21 ^d^	***	ns	***
*Subtotal (%)*	*6.36*	*5.00*	*5.04*	*6.37*	*5.97*	*7.47*	*3.89*	*4.80*			
Crotonolactone (µg/L)	127 ± 7 ^a^	245 ± 6 ^d^	262 ± 12 ^d^	251 ± 16 ^d^	200 ± 12 ^c^	158 ± 8 ^b^	345 ± 28 ^e^	159 ± 6 ^b^	***	ns	***
Butyrolactone (µg/L)	411 ± 25 ^c^	299 ± 12 ^a^	802 ± 13 ^f^	816 ± 30 ^f^	469 ± 26 ^d^	815 ± 25 ^f^	360 ± 17 ^b^	658 ± 15 ^e^	***	ns	***
Nonalactone (µg/L)	21 ± 2 ^a^	26 ± 1 ^b^	50 ± 3 ^e^	41 ± 2 ^cd^	29 ± 2 ^b^	23 ± 2 ^a^	42 ± 1 ^d^	38 ± 2 ^c^	***	ns	***
Decalactone (µg/L)	5 ± 0.1 ^b^	5 ± 0.8 ^b^	6 ± 0.4 ^c^	7 ± 0.2 ^d^	4 ± 0.1 ^a^	5 ± 0.1 ^b^	5 ± 0.3 ^b^	5 ± 0.4 ^b^	***	***	***
**Terpenes**	6 ± 0.5 ^a^	10 ± 0.6 ^b^	10 ± 1 ^b^	12 ± 0.8 ^c^	10 ± 0.5 ^b^	12 ± 1 ^cd^	14 ± 0.9 ^e^	13 ± 0.8 ^de^	***	***	***
*Subtotal (%)*	*0.07*	*0.09*	*0.04*	*0.07*	*0.08*	*0.09*	*0.07*	*0.07*			
Limonene (µg/L)	6 ± 0.5 ^a^	10 ± 0.6 ^b^	10 ± 1 ^b^	12 ± 0.8 ^c^	10 ± 0.5 ^b^	12 ± 1 ^cd^	14 ± 0.9 ^e^	13 ± 0.8 ^de^	***	***	***
**Volatile phenols**	27 ± 2 ^a^	47 ± 4 ^c^	94 ± 6 ^f^	82 ± 2 ^e^	36 ± 0.3 ^b^	60 ± 3 ^d^	125 ± 7 ^g^	54 ± 2 ^cd^	***	**	***
*Subtotal (%)*	*0.30*	*0.41*	*0.42*	*0.47*	*0.31*	*0.45*	*0.65*	*0.30*			
Guaiacol (µg/L)	6 ± 0.4 ^a^	20 ± 2 ^d^	24 ± 2 ^e^	31 ± 2 ^f^	13 ± 2 ^c^	9 ± 0.6 ^b^	30 ± 2 ^f^	19 ± 1 ^d^	***	**	*
4-vinylguaiacol (µg/L)	21 ± 2 ^a^	27 ± 3 ^a^	70 ± 4 ^d^	51 ± 2 ^c^	23 ± 1 ^a^	51 ± 3 ^c^	95 ± 7 ^e^	35 ± 2 ^b^	***	***	***
**Oak compounds**	244 ± 15 ^b^	529 ± 29 ^e^	562 ± 21 ^e^	773 ± 23 ^f^	184 ± 12 ^a^	209 ± 13 ^ab^	302 ± 15 ^c^	350 ± 12 ^d^	***	***	***
*Subtotal (%)*	*2.75*	*4.60*	*2.53*	*4.42*	*1.56*	*1.56*	*1.56*	*1.95*			
*trans*-whiskey lactone (µg/L)	54 ± 5 ^a^	113 ± 6 ^c^	132 ± 8 ^d^	155 ± 10 ^e^	91 ± 3 ^b^	109 ± 8 ^c^	159 ± 10 ^e^	178 ± 7 ^f^	***	***	***
*cis*-whiskey lactone (µg/L)	190 ± 10 ^c^	416 ± 23 ^d^	430 ± 18 ^d^	618 ± 36 ^e^	93 ± 9 ^a^	100 ± 5 ^a^	143 ± 7 ^b^	172 ± 5 ^bc^	***	***	***

*: *p* ≤ 0.05; **: *p* ≤ 0.01; ***: *p* ≤ 0.001; ns: not significant; different letters (^a, b, c, d, e, f^) show significant differences at 95% confidence level.
